# Electronic transport in two-dimensional high dielectric constant nanosystems

**DOI:** 10.1038/srep09667

**Published:** 2015-04-10

**Authors:** M. Ortuño, A. M. Somoza, V. M. Vinokur, T. I. Baturina

**Affiliations:** 1 Departamento de Física - CIOyN, Universidad de Murcia, Murcia 30071, Spain; 2 Materials Science Division, Argonne National Laboratory, Argonne, Illinois 60439, USA; 3 Novosibirsk State University, Pirogova str. 2, Novosibirsk 630090, Russia; 4 A. V. Rzhanov Institute of Semiconductor Physics SB RAS, 13 Lavrentjev Avenue, Novosibirsk, 630090 Russia

## Abstract

There has been remarkable recent progress in engineering high-dielectric constant two dimensional (2D) materials, which are being actively pursued for applications in nanoelectronics in capacitor and memory devices, energy storage, and high-frequency modulation in communication devices. Yet many of the unique properties of these systems are poorly understood and remain unexplored. Here we report a numerical study of hopping conductivity of the lateral network of capacitors, which models two-dimensional insulators, and demonstrate that 2D long-range Coulomb interactions lead to peculiar size effects. We find that the characteristic energy governing electronic transport scales logarithmically with either system size or electrostatic screening length depending on which one is shorter. Our results are relevant well beyond their immediate context, explaining, for example, recent experimental observations of logarithmic size dependence of electric conductivity of thin superconducting films in the critical vicinity of superconductor-insulator transition where a giant dielectric constant develops. Our findings mark a radical departure from the orthodox view of conductivity in 2D systems as a local characteristic of materials and establish its macroscopic global character as a generic property of high-dielectric constant 2D nanomaterials.

The unique electric properties of high dielectric constant (high-*κ*) nanosheets are the subject of the intense current attention, see Ref. 1 and references therein. High-*κ* nanosheets are of key technological importance for the fabrication of nanoscale capacitor components in high-*κ* devices. One of the central issues is the design and integration of materials that ensure robust high-*κ* properties even at thicknesses of several nanometers, allowing for a high capacitance density. Since these components are usually the largest elements in integrated circuits, reducing their size is of prime importance for the advancement of electronics. To achieve this goal it is necessary to understand the size- and related effects which are key to producing enhanced performance of thin-film capacitors and to progress in device miniaturization. There is a further class of systems, strongly disordered few nanometers thin superconducting films, whose enormous dielectric constant, developing near the superconductor-insulator transition (SIT), is crucially important. In these systems, the huge low-temperature resistance and the macroscopic quantum coherence of the so-called Cooper pair insulator, which forms at the insulating side of the SIT, are closely related to the large dielectric constant and the two-dimensional Coulomb interactions that arise as a result^2^.

One peculiarity of high-*κ* systems is that over distances not exceeding the electrostatic screening length 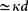, which may be quite appreciable in high-*κ* films (*d* is the film thickness), Coulomb interaction between charges at distances *d* < *r* < *κd*, acquires 2D logarithmic character^2^,^3^,^4^,^5^,^6^ with the interaction energy (between two charges *e*) approximated by^2^,^6^
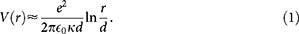
Logarithmic interaction modifies the effective density of states for hopping transport hardening the Coulomb gap^7^. Hopping conductivity then transforms from the so-called Efros-Shklovskii temperature dependence *σ* ~ exp[−(*T*_0_/*T*)^1/2^] to the Arrhenius-like thermally activated (with the logarithmic accuracy in the activation energy) behaviour 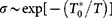, where *T*_0_ and 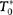 are two characteristic temperature scales of the problem. Yet the local character of the conductivity was supposed to hold. It has been recognized, however, that the most important effect of a long-range logarithmic interaction is that, since a single excess charge polarizes the whole area within the screening length, the conductivity may acquire a nonlocal character with the characteristic energy, controlling conduction, scaling as a logarithm of either the screening length or the sample size, depending on which is shorter^2^,^8^,^9^. So far this non-local behaviour was derived only on a semi-qualitative level^8^ calling for a detailed numerical investigation of hopping conductivity in the insulating high-*κ* 2D films.

We will model high-*κ* systems using the 2D capacitor network (2DCN). Such a network offers a perfect tunable laboratory for studying high-*κ* insulating sheets as it captures the tunneling nature of conductivity of the insulator and, by the proper choice of parameters, naturally generates logarithmic Coulomb interactions between charges placed at the network nodes^10^. The latter correspond to charge traps in an insulating film, and their possible random location in real systems is adequately reflected by the random character of the network. On the physical level, the effective two-dimensionality of 2DCN with respect to the electric properties is ensured by the fact that the field lines are confined to the space between the plates of the capacitors. Note that the unit cell of the 2DCN does not necessarily correspond to the unit cell of the real material, but can be rather an effective unit representing a much larger area. Furthermore, 2DCN adequately represents a Josephson junction array in the insulating state, i.e. under the condition that the charge energy of a single junction well exceeds the characteristic Josephson coupling in a single junction. Thus 2DCN also offers a perfect model for investigating electronic transport in Cooper pair insulator^8^.

We first consider an *L* × *L* square lattice shown in Fig. 1a, the two opposite edges of which are connected to the leads. The other two edges are subject to hard wall boundary conditions. The left bank is connected to the ground, while the right bank is at the potential *V*. The capacitances *C_i_*_,*j*_ assume random values drawn from the distribution *C_i_*_,*j*_ = *Ce^η^*, where *η* ∈ [−*W*/2, *W*/2]. We have chosen *C* = 1 (in units of 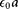, where *a* is the lattice constant) and *W* = 2. In order to take into account the leakage of field lines to outside of the 2DCN due to the finite value of *κ*, we introduce capacitances to the ground, *C*_0_. The plates of each capacitor of the 2DCN carry opposite charges and the total charge on each site *Q_i_* is the sum of the charges on the plates of the capacitors connected to it. Carriers hop from site to site transferring a quantized unity charge. Hopping over many sites with a probability that exponentially decays with the hopping distance models charge transfer in experimental systems mediated by the cotunneling mechanism. Further we consider a 2D system with randomly distributed sites, to which we will be referring as to a 2D random capacitor network (2DRCN), using periodic boundary conditions in the lateral direction. In this case the corresponding junction network without crossings can be constructed using a Delaunay triangularization algorithm^11^. Capacitors with randomly chosen capacitances were placed at the links between adjacent nodes, as shown in the Fig. 2a. Random arrays model doped thin semiconductor films and 2D strongly disordered superconducting films at the insulating side of the superconductor-insulator transition.

The total energy of the 2DCN is given by
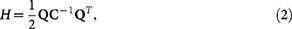
where **C** is the capacitance matrix and **Q** are vectors whose components are the charges at the corresponding nodes. The proper definition of the vectors **Q** in (2) allows inclusion of the energy due to a battery (see Methods section). The matrix **C**^−1^ ≡ **A** plays the role of an interaction matrix. Its average over disorder having the above rectangular distribution is equal to the interaction energy in the absence of disorder. In a regular array grounded via *C*_0_ capacitances, and in the continuous limit the interaction between the two charges separated by a distance *r* is proportional to the modified Bessel function *K*_0_(*r*/Λ), where the screening length is 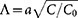. In the limit of small 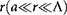 we have *K*_0_(*x*) ≈ −ln(*x*/2), and the effective interaction is given by Eq. (1) provided that we identify the screening length with Λ = *κd*, and choose 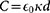 and 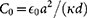. For a finite system with *L* < Λ, the effective screening length is *L*. For a 2DRCN, the average effective interaction is also logarithmic, and given by Eq. (1), but with the prefactor 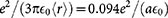, where ⟨*r*⟩ = 1.128*a* is the average length of a link which we have calculated numerically. For random nodes, *a* is defined by the relation *a* = *L*/*N*^1/2^, *N* being the number of nodes. In all cases, the unit of distance is *a* and the unit of energy is 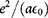. The charging energy, i.e., the energy cost for putting a unit charge at a given site, is *A_i_*_,*i*_/2. Figures 1b,c show the 3D plots of *A_i_*_,*i*_/2 for a system with *L* = 50 as functions of the coordinates, *x_i_* and *y_i_*. Figure 1b corresponds to the situation where 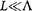, and Fig. 1c shows the energy distribution for the opposite limiting case 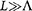. The electrodes screen the interaction so that the charging energy near them is small. By contrast, near the free banks, where we assume hard wall boundary conditions, the charging energy is large. The charging energy at the center of the sample is roughly equal to one half of the energy given by equation (1) with *r* ≈ 0.2.

Hopping conductivity *σ* is determined by the charge transfer rate between sites *i* and *j*
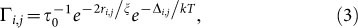
where 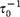 is the phonon frequency, of the order of 10^13^ 14;Hz, *r_i_*_,*j*_ the hopping distance, *ξ* the localization length and Δ*_i_*_,*j*_ the transition energy. To simulate hopping conductivity in the system of interacting electrons, we employ a kinetic Monte Carlo method^12^,^13^. It is customary in the simulations of this kind to produce the electric current via imposing on the system a uniformly distributed electric field. Such an approach, however, is not adequate in our case. Thus, we employ a scheme corresponding to the experimental situation, where the electric current is generated by the potential difference between the leads at the opposite sides of the sample. For a given sample we calculate conductivity from the dissipated electric power. Finally, we carry out averaging of ln *σ* over the set of samples (an ensemble averaging). Hereafter we will imply averaged quantity for the logarithm of conductivity, ln *σ*.

The logarithm of conductivity, ln *σ*, versus 1/*T* for regular 2DCN with Λ → ∞, is shown in Fig. 1d. We see that at high temperatures, *T* > 0.016, conductivities for 2DCN of different sizes are nearly the same, but start to appreciably differ from each other with separations growing upon cooling down. Thus, the set of ln *σ* versus 1/*T* curves acquires a fan-like shape at lower temperatures when crossing over to Arrhenius-like temperature behaviour, *σ* ∝ exp(−*E*_ac_/*T*), where *E*_ac_ is the activation energy. Dependence *E*_ac_(*L*) extracted from the slopes of ln *σ* vs 1/*T* plots is displayed in Fig. 1e. To extrapolate the data taken on finite samples to the infinite size system we plotted ln *σ* taken at given temperature as function of 1/*L*, see Fig. 1f. The points corresponding to the infinite system are marked by stars in Figs. 1d,f. The envelope solid line in Fig. 1d is a fit of these extrapolation data by the formula
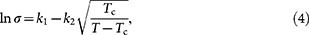
describing conductivity in the critical region just above the Berezinskii-Kosterlitz-Thouless (BKT) transition. Here *k*_1_ = 2.48 and *k*_2_ = 1.65, and *T*_c_ = 0.0155 is the BKT transition temperature.

In the case where 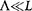 the logarithmic size scaling resulting from logarithmic Coulomb interaction manifests itself as Λ-dependence of the activation energy in accord with the conjecture of Ref. 8. Shown in the Fig. 1g,h are the ln *σ* vs 1/*T* plots for systems with different Λ and the corresponding *E*_ac_(Λ) inferred from the Arrhenius fits. A few comments are in order. First, to summarize, the magnitudes of the characteristic activation energies *E*_ac_ are of the order of the charging energy at the center of the sample and increase as *E*_ac_ = *E*_0_ ln(min{*L*, Λ}), as shown in Figs. 1e,h, where 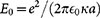. Second, there are deviations from the pure logarithmic scaling. In the case Λ < *L* this deviation occurs at largest Λ, see Fig. 1h, where it compares to the system size. In the situation 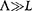, the energy *E*_ac_(*L*) fails to adhere to the logarithmic scaling at small sizes, see Fig. 1e. The reason is that in the neutral system of positive and negative charges placed on a square grid, the genuine BKT transition interferes with the ‘antiferromagnetic’ order transition at low temperatures where the system falls into a configuration with the alternating plus and minus charges^3^,^14^. To ensure the presence of antiferromagnetic order, we simulated regular systems with periodic boundary conditions and checked that the results strongly depend on the even/odd character of the transversal dimension.

To eliminate the antiferromagnetic order transition we have simulated conductivity in a network with a random spatial distribution of the nodes, see Fig. 2a. We investigate two-dimensional random capacitor network (2DRCN) in the absence of capacitances to the ground corresponding to infinite screening lengnth. The corresponding ln *σ* vs. 1/*T* dependences are displayed in Fig. 2b. These curves are pretty similar to those for regular lattices; the red envelope curve also obeys Eq. (4) evidencing the pure BKT transition at *T*_c_ = 0.019. Near and below the BKT transition, the conductivity displays an Arrhenius-like behaviour. As for regular lattices, the characteristic activation energy *E*_ac_ is of the order of the charging energy at the center of the sample. A closer look at the activated behaviour reveals that in smaller systems a change in the slope (flattening) of the Arrhenius dependences at *T* ≈ 0.015 takes place, indicating a possible change in the conduction mechanism and the corresponding reduction of *E*_ac_ at low temperatures. The activation energies *E*_ac_ as functions of ln *L* are displayed in Fig. 2c. Gray squares correspond to regular network given for comparison, colored squares represent 2DRCN activation energies at moderate temperatures, while open diamonds stand for the low-temperature parts of the ln *σ* vs. 1/*T* curves. The lines are linear fits showing that the activation energy nicely adhers to logarithmic spatial scaling behaviours in both temperature regions since the antiferromagnetic ground state is destroyed by disorder. The slope of the moderate temperature *E*_ac_(*L*)-dependence labeled as (1) is twice as large compared to that of the low temperature region labeled as (2) on Fig. 2c. The low-temperature decrease of slopes of ln *σ*(*T*) signals that processes with the lower activation energy become important and indicates that at low temperatures dissociation of the pairs composed of single-particle excitations and their images, as predicted in Ref. 15, may noticeably contribute to transport, whereas at intermediate *T* the leading contribution is due to splitting of the bound pairs in the bulk.

To gain better insight into the nature of the conducting state of the 2DRCN, it is instructive to visualize the distribution of active charges contributing to electronic transport. As there are no offset charges, all the sites are neutral at equilibrium at *T* = 0. As temperature increases some sites become charged. Panels (d–f) of Fig. 2 show the snapshots of charge distributions at three different representative temperatures for the system with *L* = 50. The grey bars are the leads that are connected to the left and right edges, while periodic boundary conditions are imposed between the top and bottom edges. Black dots correspond to neutral sites, while blue and red circles depict positively and negatively charged sites, respectively. The snapshot at panel (d) is taken well above the BKT transition, at *T* = 0.04. The panel (e) represents the snapshot from the critical region slightly above the transition, at *T* = 0.02. Finally, panel (f) displays charge distribution at *T* = 0.015, which is well below *T_c_* of the infinite system. As expected, at the lowest temperature the positively and negatively charged sites appear very close to each other as pairs, since charges of the opposite signs tend to be bound below the BKT transition. This demonstrates a remarkable feature of hopping conductivity in electronic glass, namely that only a small fraction of the sites takes part in the transport process. When *T* increases more pairs appear and at about the transition temperature the charged sites cannot be presented as a set of pronounced pairs anymore, again in complete agreement with the expectations: near the BKT transition the distance between the pairs compares with the typical pair size.

Figures 1 and 2 represent the main result of our work. We find that as a direct consequence of the logarithmic interaction between the charges in 2D networks of capacitors (i) the characteristic energy governing the Arrhenius behaviour of conductivity scales as ln(min{Λ, *L*}) proving the conjecture of Refs. 8, 9. We further find that (ii) this logarithmic scaling of activation energy develops near and below the BKT transition. Finally, (iii) we demonstrate two distinct regimes of hopping transport, the moderate-temperature hopping characterized by local conductivity and low-temperature transport governed by non-local conductivity stemming from the global Coulomb blockade effects.

To cross check the existence of the low-temperature BKT phase we inspect the nonlinear behaviour of the conductivity of 2DRCN of different sizes. Shown in Fig. 3a is ln *σ* vs. ln *F*, where *F* = *V*/*L* is the applied electric field, for several system sizes at *T* = 0.018. The red stars correspond to extrapolation of the data to the infinite system. The straight line describes the *σ*(*F*) dependence in the BKT phase of the infinite system and is given by
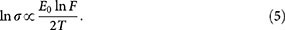
due to dissociation of the bound pairs by applied electric field.

The benchmark of the BKT transition is the power-law behaviour of the current-voltage dependence *I* ∝ *F ^α^* with *α* = 1 + *E*_0_/(2*T*) below the BKT transition, and *α* = 3 at *T*_c_. We present in Fig. 3b the ln *I* vs ln *F* plots shown by colored diamonds in the high field region for four temperatures below the transition for the system with the size *L* = 50. The dotted lines correspond to the expected BKT power-law behaviour. The only fitting parameter is the prefactor in Eq. (5). We see an excellent agreement for data taken at three lowest temperatures. The solid straigh lines are fits by *I* ∝ *F ^α^*. The values of *α* inferred from these dependences are plotted as function of 1/*T* in Fig. 3c. The straight line is the linear fit. The value of *T*_c_ obtained by extrapolation of this fit to *α* = 3 is close to 0.019 marked by the vertical stroke in the panel (c) and found above from the analysis of linear conductivity (see Fig. 2b).

In conclusion, we revealed that high-*κ* thin films exibit striking nonlocal conductivity. Long range logarithmic Coulomb interactions give rise to logarithmic dependence of the hopping activation energy upon the minimum of either the system size or dielectric screening length. On a qualitative level the origin of the logarithmic size dependence of the activation energy can be understood as follows. The conductance of the system is 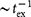, where *t*_ex_ is the time of the first exit from the system of a freely diffusing electron, implying *t*_ex_ ~ exp(*V*(*L*)/*k*_B_*T*), since *V*(*L*) is the energy necessary for separating two charges of the opposite signs over either the system size or screening length whichever is smaller. Hence *E*_ac_ ~ ln min{Λ, *L*}. This novel functionality is of crucial importance to electronic applications of these materials. The obtained results call for rethinking of and form the base for understanding fundamental features of Coulomb systems^16^ including slow relaxation, memory effects and aging^3^,^17^. It is furthermore imperative to revisit data on hopping conductivity in high-*κ* sheets like barium titanate ceramics, exhibiting colosal permittivity^18^, graphene oxide and titanium oxide nanosheets^19^, and Bi_2_Sr_2_Co_2_O_8_ nanosheets^20^. The capacitor network model is a perfect laboratory for studying properties of Josephson junction arrays and disordered superconducting films. Our findings offer a firm basis for understanding the sample size logarithmic scaling of characteristic energies of tunneling conductivity observed at the insulating side of the superconductor-insulator transition in strongly disordered superconducting films^2^,^8^, as a result of the global Coulomb blockade.

## Methods

We employ a kinetic Monte Carlo method to calculate the conductivity of 2DCN. We consider the Hamiltonian given by equation (2) where the components of **Q** are the charges 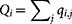 of the nodes in the sample, where *q_i_*_,*j*_ is the charge in the plates of the capacitor connecting nodes *i* and *j*, satisfying
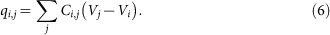
For the nodes in the leads it is convenient to define the components of **Q** as *C_j_*_,*i*_*V*, where *V* is the potential at the lead of node *i*, and *C_j_*_,*i*_ the capacitance connecting *i* with a node *j* in the sample. Thus, the vector **Q** has dimension *L* × (*L* + 1)
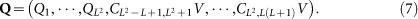
This vector is related to the vector of the components of the potential
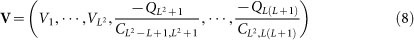
through the relation
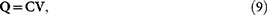
where the capacitance matrix **C** has components equal to
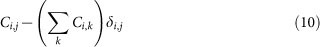
between nodes in the sample, and equal to *C_i_*_,*i*+*L*_ for the diagonal term of node *i* on the lead and for the off-diagonal term connecting this site with its neighbor in the sample. It is easy to prove that with this definition the Hamiltonian given by (2) is equivalent to
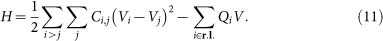
The inclusion of the components of **Q** in the leads takes into account the energy provided by the battery and tilt the electric potential along the longitudinal direction *y*. It is essential to include the effects of the electric field *F* = *V*/*L* through a realistic procedure, such as the previous one, and not through a local change −*eFy_i_*_,*j*_ in the hopping energy, which is standard procedure in electron glass simulations^13^, since the latter washes out global charging effects^21^.

The employed Monte Carlo algorithm deals with single-electron transitions with transition rates given by equation (3). At each Monte Carlo step, the algorithm chooses a pair of sites (*i*, *j*) with the probability proportional to exp(−2*r_ij_*/*ξ*), Ref. 12. It first checks whether the transfer of a unit charge from site *i* to *j* is compatible with the restrictions of the model. Then it calculates the transition energy
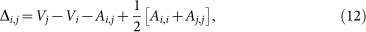
where 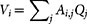 is the potential at *i*, and the hop is performed when Δ*_i_*_,*j*_ is negative or with probability exp(−Δ*_i_*_,*j*_/*T*) otherwise. All site potentials *V_i_* are recalculated after every successful transition. The time step associated with a hop attempt is 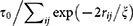, where *τ*_0_ is the inverse phonon frequency. The last term in (12) is the charging energy of the nodes involved and is not present in Coulomb gap calculations. For sites at the leads the charging energy is assumed to be zero.

The algorithm starts from an initial charge configuration and follows the dynamics at a given temperature, monitoring all relevant magnitudes, among others the energy absorbed or emitted in the process. The allowed node charges are either ±1/2, or 0 and ±1, both cases producing similar results. Once the system is in a stationary situation, the conductivity of each sample is calculated from the dissipated power and its logarithm is averaged over the samples. The number of Monte Carlo steps performed in this calculation drastically increases with decreasing *T*, and it is determined by the condition that the net charge crossing one of the leads is on the order of 1000. The number of the samples in the set varies from 1000 for the smaller size to 200 for the largest size.

## Figures and Tables

**Figure 1:**
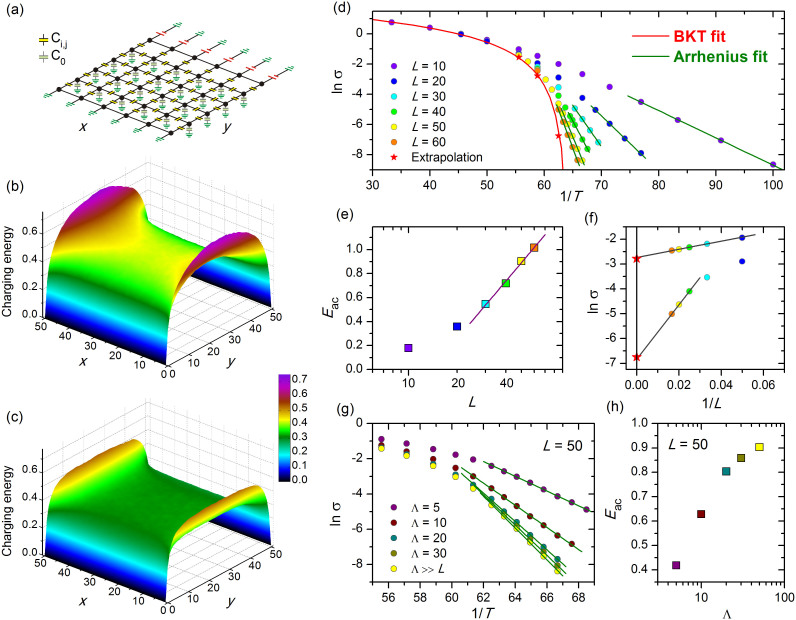
Two dimensional regular network of capacitors (2DCN). (a) A sketch of a square network of capacitors that models electric properties of an insulating high-*κ* nanosheet. Capacitors connecting the nodes have capacitances {*C_i_*_,*j*_}; nodes are linked to the ground by capacitors with capacitances to the ground {*C*_0_}. The electrostatic screening length 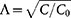. (b) Three dimensional plot of the electrostatic energy of the excess charge in the network with the size *L* = 50 and in the absence of capacitances to the ground, i.e. with Λ → ∞. The network is connected to the leads at *y* = 0 and *y* = 50. (c) The same as (b) but with 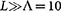. The color scales is the same for both (b) and (c). (d) Logarithm of conductivity *σ*, shown by colored circles, as function of the inverse temperature, 1/*T*, for different system sizes listed in the legend and Λ → ∞. Straight solid lines are Arrhenius fits, *σ* ∝ exp(−*E*_ac_/*T*). The BKT red curve is given by Eq.(4) and fits the ln *σ*(1/*T*) values (shown by stars) obtained by extrapolation of the system size to infinity. (e) Size dependence of the activation energy *E*_a*c*_ inferred from the Arrhenius fits of panel (d). (f) Plots of ln *σ* vs. 1/*L* for temperatures *T* = 0.016 (lower plot) and *T* = 0.017 (upper plot) giving conductivities for the infinite-size systems. The legend for symbols is the same as that in the panel (d). (g) Logarithm of conductivity *σ*, shown by colored circles, as function of the inverse temperature, 1/*T*, for different Λ listed in the legend for *L* = 50. Straight lines are Arrhenius fits. (h) Activation energies inferred from panel (g) as functions of Λ. The last yellow point depicts Λ = *L* = 50. The error bars in panels (d–h) are smaller than the size of the symbols and have not been shown.

**Figure 2:**
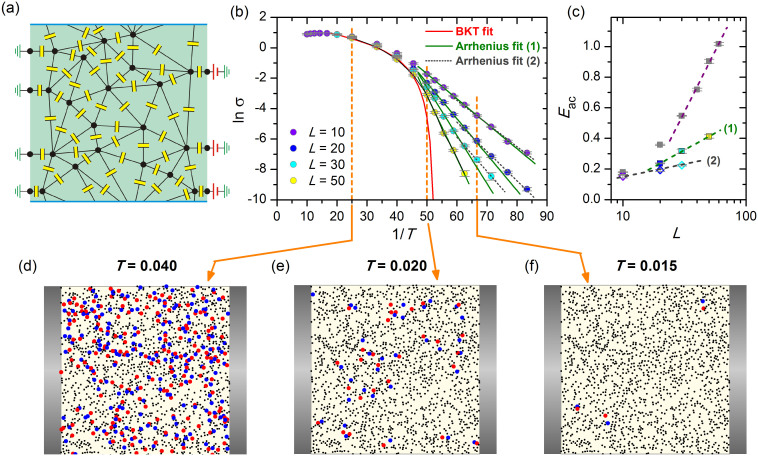
Two-dimensional random capacitor network (2DRCN). (a) A sketch of 2DRCN. The electrode connected to the left edge row of capacitors is grounded, the electrode at the right edge has potential *V*. (b) Logarithm of conductivity *σ*, shown by colored circles, as function of the inverse temperature, 1/*T*, for different system sizes listed in the legend and Λ → ∞. Straight solid lines are Arrhenius fits (1), *σ* ∝ exp(−*E*_a*c*_/*T*), in the temperature interval 0.020 > *T* > 0.016. The dashed lines are Arrhenius fits (2) for *T* < 0.016. The red curve represents the BKT behaviour given by Eq.(4) extrapolated from the data at temperatures *T* > 0.020. (c) Size dependence of the activation energy *E*_a*c*_ inferred from the Arrhenius fits (1) and (2) of panel (d). The upper grey symbols replot the data from Fig. 1e for the regular network presented for comparison. (d)–(f) Snapshots of the charge distribution corresponding to three representative temperatures marked by vertical dashed lines in the panel (b). Black dots stand for neutral sites. Blue and red circles depict the sites with the excess negative and positive charges at the site, respectively.

**Figure 3:**
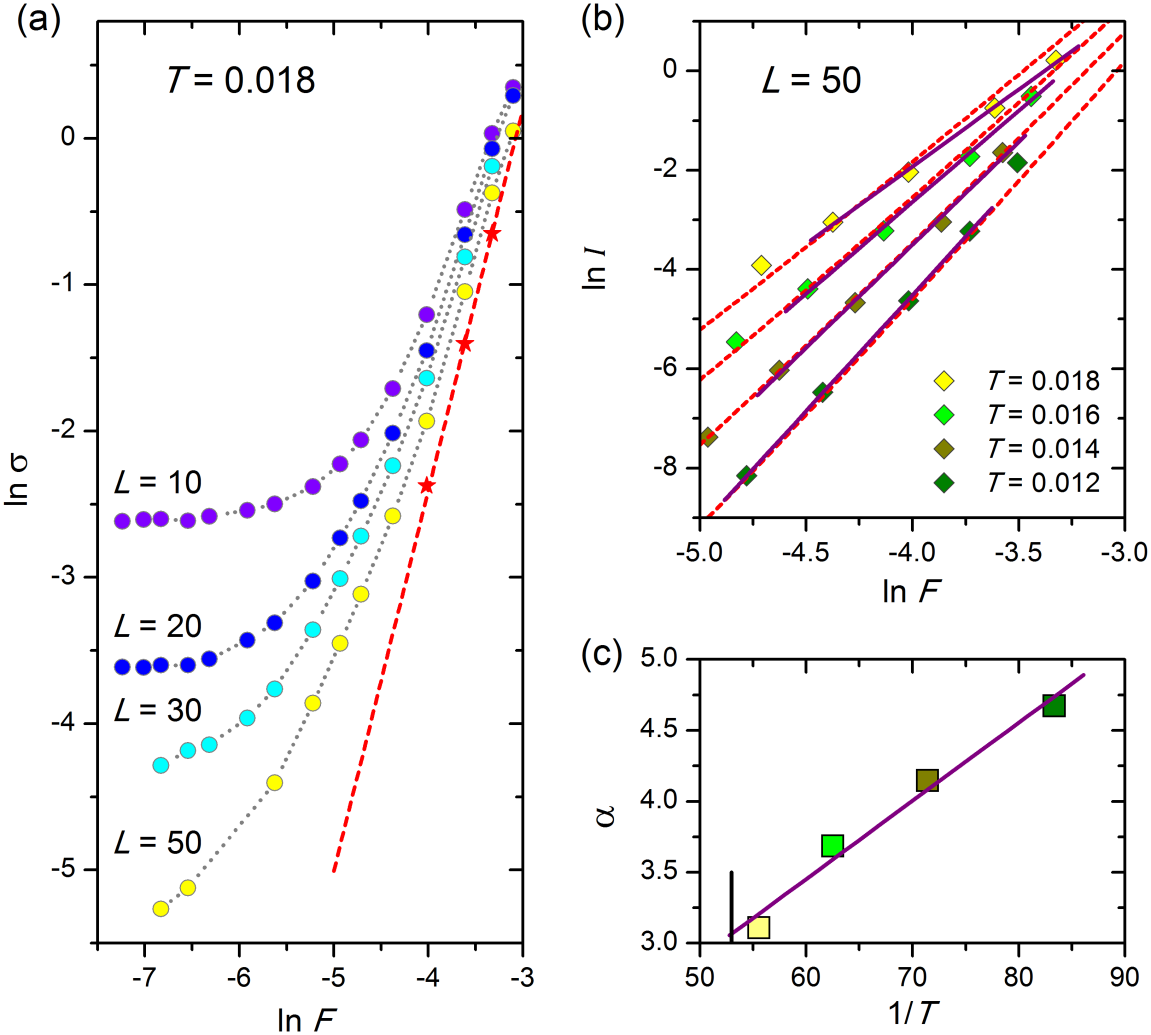
Nonlinear current-voltage characteristics for 2DRCN. (a) Plots of conductivity as a function of the electric field *F* on a double-logarithmic scale at *T* = 0.018 for several values of *L*, indicated in the figure. The red stars are obtained by extrapolation to infinite system size via the same procedure as shown in Fig. 1f. Straight line is given by Eq. (5) and describes conductivity due to dissociation of the bound electron-hole pairs. (b) Plots of the current, *I*, as a function of the electric field on a double-logarithmic scale for several values of *T* indicated in the figure and *L* = 50. The dotted straight lines are the nonlinear *I*-*F* curves corresponding to conductivity from Eq. (5). The fitting parameter is the prefactor in Eq. (5). The solid straight lines are fits according to formula *I*∝*F ^α^*. (c) Plot of *α* versus 1/*T*. The straight line is a linear fit; the vertical stroke marks *T*_c_ given by the BKT fit of linear conduction data in Fig. 2b.
